# Perceptual “Read-Out” of Conjoined Direction and Disparity Maps in Extrastriate Area MT

**DOI:** 10.1371/journal.pbio.0020077

**Published:** 2004-03-16

**Authors:** Gregory C DeAngelis, William T Newsome

**Affiliations:** **1**Department of Anatomy and Neurobiology, Washington University School of MedicineSt. Louis, MissouriUnited States of America; **2**Howard Hughes Medical Institute and Department of Neurobiology, Stanford University School of MedicineStanford, CaliforniaUnited States of America

## Abstract

Cortical neurons are frequently tuned to several stimulus dimensions, and many cortical areas contain intercalated maps of multiple variables. Relatively little is known about how information is “read out” of these multidimensional maps. For example, how does an organism extract information relevant to the task at hand from neurons that are also tuned to other, irrelevant stimulus dimensions? We addressed this question by employing microstimulation techniques to examine the contribution of disparity-tuned neurons in the middle temporal (MT) visual area to performance on a direction discrimination task. Most MT neurons are tuned to both binocular disparity and the direction of stimulus motion, and MT contains topographic maps of both parameters. We assessed the effect of microstimulation on direction judgments after first characterizing the disparity tuning of each stimulation site. Although the disparity of the stimulus was irrelevant to the required task, we found that microstimulation effects were strongly modulated by the disparity tuning of the stimulated neurons. For two of three monkeys, microstimulation of nondisparity-selective sites produced large biases in direction judgments, whereas stimulation of disparity-selective sites had little or no effect. The binocular disparity was optimized for each stimulation site, and our result could not be explained by variations in direction tuning, response strength, or any other tuning property that we examined. When microstimulation of a disparity-tuned site *did* affect direction judgments, the effects tended to be stronger at the preferred disparity of a stimulation site than at the nonpreferred disparity, indicating that monkeys can selectively monitor direction columns that are best tuned to an appropriate conjunction of parameters. We conclude that the contribution of neurons to behavior can depend strongly upon tuning to stimulus dimensions that appear to be irrelevant to the current task, and we suggest that these findings are best explained in terms of the strategy used by animals to perform the task.

## Abstract 2

Greg DeAngelis and Bill Newsome use microstimulation to activate small populations of neurons with different combinations of response properties in monkey visual cortex

## Introduction

Determining how information is “read out” of sensory maps in the cerebral cortex is of fundamental importance for understanding how neural activity gives rise to cognitive processes such as perception, planning for action, and working memory. A substantial portion of our knowledge about sensory read-out comes from studies of the middle temporal (MT) visual area, an extrastriate area known to play important roles in processing visual motion information (for reviews, see Maunsell and Newsome 1987; Albright 1993; Andersen 1997). The vast majority of MT neurons are directionally selective (Zeki 1974), and they are arranged in an orderly system of direction columns that run perpendicular to the cortical surface (Albright et al. 1984; Malonek et al. 1994). In addition, most MT neurons are also selective for binocular disparity (Maunsell and Van Essen 1983; DeAngelis and Uka 2003), and these neurons are organized in a topographic map of disparity preference. Regions of strong disparity selectivity are intercalated among patches of MT neurons with weak disparity tuning, and these strongly tuned regions contain a set of disparity columns that are interwoven with the direction columns (DeAngelis and Newsome 1999).

Understanding how information is read out of cortical structures is complicated by the existence of topographic maps for multiple stimulus dimensions or features within a single area, such as those in MT and many other sensory areas of the cortex (Mountcastle 1997). For example, several studies have shown that electrical microstimulation of direction columns in MT can influence perceptual judgments of visual motion during the performance of a direction discrimination task (Salzman et al. 1990, 1992; Murasugi et al. 1993; Salzman and Newsome 1994; Bisley et al. 2001; Nichols and Newsome 2002), and, similarly, that microstimulation of disparity columns can influence perceptual judgments of depth (DeAngelis et al. 1998). In all of these studies, however, the presence and size of the microstimulation effects were highly variable from experiment to experiment, suggesting that the read-out mechanism is more complex than is presently understood. Notably, each of these studies concentrated on a single physiological property—the one of direct relevance to the task at hand—in selecting MT sites for microstimulation experiments (direction tuning for direction discrimination tasks, and disparity tuning for depth discrimination tasks). Potential effects of tuning to multiple stimulus parameters on the read-out mechanism were largely ignored.

We therefore designed the current study to ask two specific questions concerning the interaction of direction and disparity tuning in motion perception. (1) Do MT columns that possess or lack disparity tuning contribute differentially to direction judgments? We used electrical microstimulation to test the hypothesis that neurons in the nondisparity-selective regions of MT contribute to motion perception, whereas those in the disparity-selective regions are mainly involved in depth perception. Our hypothesis was confirmed for two of the three monkeys in this study: microstimulation of nondisparity-selective sites produced strong direction biases, whereas stimulation of disparity-selective sites had little or no effect. For the third monkey, microstimulation biased direction judgments when it was applied at either disparity-selective or nonselective sites. For disparity-tuned sites that did yield effects on direction judgments, we also asked a second question. (2) Does the influence of a disparity-tuned column on direction judgments vary as a function of the actual disparity of the motion display? We found that stimulation effects were stronger when the disparity of the visual stimulus matched the preferred disparity of the stimulated column.

We conclude that tuning for task-irrelevant stimulus dimensions can exert dramatic effects on the contribution of cortical neurons to a particular perceptual judgment. In extreme cases, columns tuned for an irrelevant dimension (disparity) fail to contribute at all to perceptual judgments of the task-relevant dimension (direction). In less extreme cases, the contribution of a column is modulated by tuning along the task-irrelevant dimension, so that microstimulation effects are obtained primarily when the visual stimulus possesses the right conjunction of properties (direction and disparity) to excite the column optimally. We discuss our findings in terms of the strategies employed by animals to solve the task.

## Results

**Figure 1:**
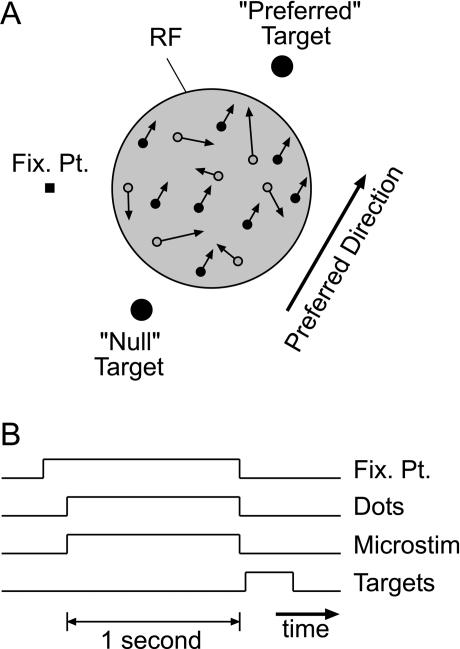
Behavioral Task Used to Assess the Effects of Microstimulation on Direction Discrimination Performance (A) Schematic depiction of the visual stimulus display, showing the FP, the preferred and null response targets, and a variable-coherence random-dot pattern presented within the MU RF of MT neurons. An adjustable fraction of the dots (signal dots, filled circles) moved in the preferred or null direction of the MT neurons, while the remaining dots (noise dots, open circles) were randomly replotted on each refresh of the display, thus creating a masking motion noise. Signal and noise dots could be presented at a range of binocular disparities. Outside the MU RF, the remainder of the visual display was filled with zero-disparity, stationary dots (not shown). (B) Sequence of trial events in the microstimulation experiment. During each trial, the FP appeared first. Roughly 300–500 ms after the monkey achieved fixation, the random-dot pattern appeared in the MU RF. On half of the trials, selected at random, microstimulation was turned on during the visual stimulus. After a 1-s viewing period, dots and microstimulation were extinguished, and the two small target disks appeared. The animal was rewarded for making a saccade to the target corresponding to the direction of motion of the signal dots.

Microstimulation experiments were performed at 102 recording sites in area MT of three rhesus monkeys (38 sites in monkey S, 36 sites in monkey T, and 28 sites in monkey R) during the performance of the direction discrimination task illustrated in Figure 1 (see Materials and Methods for details). The results are presented in three sections. First, we examine how the effects of microstimulation depend on the strength of disparity tuning at the stimulation site. Second, we present control analyses to exclude trivial explanations for the dependence of microstimulation effects on disparity-tuning strength. Third, for sites where the multiunit (MU) activity exhibited moderate to strong disparity selectivity, we examine whether the effect of microstimulation on direction judgments depends on the disparity at which the visual stimulus is presented.

### Relationship between Efficacy of Microstimulation and Disparity Selectivity

We have previously shown that disparity-selective neurons tend to occur within discrete patches of MT (DeAngelis and Newsome 1999). Given this patchy distribution, we asked whether disparity-selective and nonselective patches of MT contribute equally to performance on the direction discrimination task. In all cases, the disparity of the visual stimulus was chosen to elicit a near-maximal response from MU activity at the stimulation site. Also, because microstimulation was only attempted in portions of electrode penetrations where direction selectivity was consistently near-maximal (see Materials and Methods), all experiments were done at MU recording sites with strong direction tuning.

**Figure 2:**
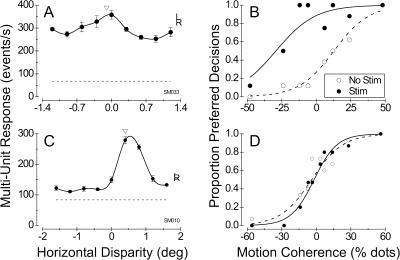
Effect of Microstimulation on Direction Judgments at Two Illustrative Stimulation Sites from Monkey S A site with weak disparity tuning (DTI = 0.37) is shown in (A) and (B) and a site with strong disparity tuning (DTI = 0.87) is shown in (C) and (D). (A) Disparity tuning of MU activity at a stimulation site with weak disparity selectivity. Filled circles show the mean response to four stimulus presentations at each disparity, with error bars indicating ±1 SE. The solid curve is a cubic spline interpolation. The letters “L” and “R” are plotted at the response levels obtained when the same stimulus is shown only to the left and right eyes, respectively. The dashed horizontal line gives the spontaneous activity level in the absence of any visual stimulus, and the arrowhead denotes the disparity chosen for the direction discrimination task. (B) Effect of microstimulation on direction judgments for the site with the disparity tuning indicated in (A). The proportion of decisions made by the monkey toward the neurons' preferred direction of motion is plotted against the motion coherence of the random-dot stimulus. Open circles show the behavior obtained in the absence of microstimulation; the dashed curve is the best fit to these data using logistic regression. Filled circles and the solid curve show data from randomly interleaved trials in which microstimulation was applied. Note the large leftward shift of the psychometric function, equivalent to 38.7% dots (logistic regression, *p* < 0.001). (C) Disparity tuning of MU activity at a stimulation site with strong disparity selectivity. Again, the arrowhead denotes the disparity at which dots were presented in the direction discrimination task. (D) Effect of microstimulation on direction judgments for the site with the disparity tuning indicated in (C). In this case, there was no significant shift of the psychometric function when microstimulation was applied (*p* > 0.5); the small difference in slope between stimulated and nonstimulated trials is also not significant (*p* > 0.25).

Figure 2 shows data from two illustrative experiments performed on monkey S. Figure 2A shows the disparity tuning of MU activity at a stimulation site with modest disparity selectivity. Based on this tuning curve, we chose a small near disparity of −0.1° for the random-dot stimuli used in the direction discrimination task (arrowhead in Figure 2A). Microstimulation at this weakly tuned site strongly biased the monkey's decisions toward the preferred direction of motion (Figure 2B). The net effect of this bias was a large leftward shift of the psychometric function (equivalent to 38.7% dots; logistic regression, *p* << 0.001), with no significant change in the slope of the curve (logistic regression, *p* > 0.5). This effect is qualitatively similar to those obtained previously in our laboratories (e.g., Salzman et al. 1992; Murasugi et al. 1993).

Figure 2C shows MU responses for a stimulation site with strong disparity selectivity. Activity at this site exhibited a clear preference for far disparities, and we chose a disparity of 0.4° for the direction discrimination task. Despite the fact that dots were presented at the preferred disparity and MU activity was strongly direction selective (data not shown), microstimulation had no significant effect on the monkey's judgments (Figure 2D; logistic regression, *p* > 0.5 for shift, *p* > 0.25 for slope). Thus, the activity of neurons at this stimulation site did not appear to contribute to direction discrimination.

**Figure 3:**
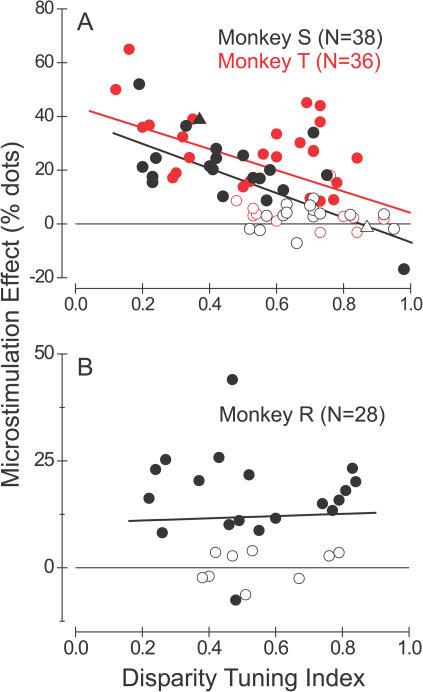
Relationship between the Efficacy of Microstimulation and the Strength of Disparity Tuning Each datum represents one experiment, with filled symbols denoting significant effects of microstimulation (logistic regression, *p* < 0.05). The vertical axis shows the leftward shift of the psychometric function induced by microstimulation. Thus, positive values correspond to shifts toward the preferred direction of motion. The horizontal axis shows the DTI for MU activity at each stimulation site. (A) Data for monkey S (black symbols, *n* = 38) and monkey T (red symbols, *n* = 36). For both animals, there is a highly significant tendency for the effect of microstimulation to decline with increasing disparity selectivity (linear regression, *r* = −0.69 for monkey S, *r* = −0.52 for monkey T, *p* < 0.001 for both). The black, filled triangle denotes the experiment depicted in Figure 2A and 2B; the black, open triangle corresponds to the experiment of Figure 2C and 2D. (B) Data for monkey R (*n* = 28). In this case, the two variables are uncorrelated (*r* = −0.025, *p* > 0.9).

Figure 3A summarizes results from 38 similar experiments performed in monkey S (black symbols) and 36 experiments in monkey T (red symbols). The effect of microstimulation on direction judgments is plotted against the Disparity Tuning Index (DTI) of MU activity at each stimulation site. DTI values near 1.0 indicate very strong disparity selectivity, whereas values near 0.0 denote poor tuning (see Materials and Methods, Equation 2). Filled symbols denote statistically significant shifts of the psychometric function due to microstimulation (logistic regression, *p* < 0.05), whereas open symbols indicate nonsignificant effects. The filled and open triangles correspond to the examples shown in Figure 2B and 2D, respectively. For both monkeys, the data reveal a strong negative correlation between the magnitude of the stimulation effect and the DTI of MU activity (linear regression, monkey S, *r* = −0.69, *n* = 38; monkey T, *r* = −0.52, *n* = 36; *p* << 0.001 for both animals). An analysis of covariance that included monkey identity as a coregressor revealed no significant difference between regression slopes for the two animals (ANCOVA, *p* > 0.6). Note that microstimulation almost always produced a significant effect on direction judgments in experiments for which the DTI was less than 0.5. In contrast, significant effects of microstimulation occurred much less frequently when the DTI exceeded 0.5.

The result in Figure 3A is interesting for two main reasons. First, it suggests that a substantial amount of variance in the efficacy of microstimulation may be accounted for by the disparity tuning of neurons at the stimulation site. This may explain why previous microstimulation studies reported a large number of nonsignificant effects (e.g., Salzman et al. 1992; Murasugi et al. 1993). In those studies, the disparity tuning of activity at stimulation sites was not measured, and all stimuli were presented at zero disparity. Second, this result is interesting because it suggests that monkeys S and T may read out activity from MT in a manner that is highly dependent on the functional architecture for binocular disparity. In formulating decisions about motion direction, these animals appeared to rely most heavily on direction-selective columns that were nonselective for disparity. In contrast, columns that were strongly tuned for disparity exerted substantially less influence on the animals' decisions. We shall address possible explanations for this finding in the Discussion.

We obtained quite different results in a third animal, monkey R (Figure 3B). For this animal there was no significant correlation between the strength of the microstimulation effect and the DTI (*r* = −0.025, *p* > 0.9, *n* = 28). We often observed significant effects of microstimulation at sites with strong disparity tuning. It is worth emphasizing that all of the data in Figure 3 were collected using a near-optimal stimulus disparity. Thus, monkey R's decisions were usually biased by microstimulation of any direction column that was strongly activated by the visual stimulus. Effects of microstimulation at nonoptimal stimulus disparities will be addressed in a later section.

The individual differences between monkeys in the data of Figure 3 may reflect different strategies used by the animals to extract motion information from area MT. Under the conditions of our task, it appears that monkeys S and T relied predominantly on direction columns with poor disparity tuning, whereas monkey R seemed also to utilize motion signals carried by regions of MT with strong disparity selectivity. In principle, this difference in strategy might have allowed monkey R to perform better on the task, as he could pool MT responses over a larger population of neurons. To examine this possibility, we analyzed the monkeys' behavioral data from trials when microstimulation was turned off, and we computed a psychophysical threshold for each stimulus disparity in each experiment (see Britten et al. 1992 for methodological details). Interestingly, we found that the mean psychophysical threshold for monkey R (16.1% ± 1.2% standard error [SE], *n* = 51) was significantly lower than the mean psychophysical thresholds for monkey S (21.5% ± 0.9% SE, *n* = 89) and monkey T (22.8% ± 1.0% SE, *n* = 70) (Student's t-test, *p* < 0.0005 for both comparisons). In contrast, the average slope of the psychometric functions did not differ between the three animals (ANOVA, *p* > 0.7). We shall consider these issues further in the Discussion.

### Functional Segregation of the Perceptual Effects of Microstimulation

**Figure 4:**
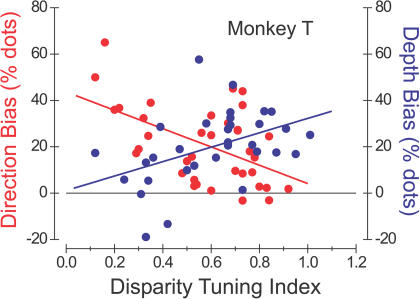
Effects of Microstimulation on Direction Discrimination and Depth Discrimination for One Animal (Monkey T) That Was Tested in Both Tasks Plotted as a function of DTI, red circles indicate the horizontal shift of the psychometric function induced by microstimulation during the direction discrimination task with stimuli at the preferred disparity for each site (left axis). These data, along with the best linear fit (solid line), are replotted from Figure 3A. Blue circles denote the effects of microstimulation during a depth discrimination task with stimuli at the preferred direction of motion for each site (right axis; data from DeAngelis et al. 1998). The dashed line shows the best linear fit to these data (*r* = 0.45, *p* = 0.01, *n* = 32).

Monkeys T and R were subjects both in the current set of experiments and in a separate study in which we showed that stimulation of disparity-tuned columns influences perceptual judgments of depth (DeAngelis et al. 1998). For these animals, therefore, we were able to compare directly how the strength of microstimulation effects in these two tasks depended on the disparity selectivity of the stimulation sites. Figure 4 shows, for monkey T, the strength of the microstimulation effects in the direction discrimination task (red symbols, reproduced from Figure 3A) and in the depth discrimination task (blue symbols, *r* = 0.45, *p* = 0.01, *n* = 32) as a function of the DTI. The data reveal a clear inverse relationship between the two effects. Columns with low DTIs produce large effects on direction discrimination performance and little or no effect on depth discrimination. In contrast, columns with large DTIs show the converse pattern. In this monkey, therefore, the functional segregation of MT columns according to the strength of disparity tuning is particularly clear.

It is important to note that the differences between animals seen in Figure 3 cannot be explained by any training experience involving the depth discrimination task. The present experiments were completed before any of the animals were subsequently trained to perform the depth discrimination task.

### Excluding Alternative Explanations for Dependence of Microstimulation Effects on Disparity Selectivity

The striking result in Figure 3A could be explained trivially if disparity-tuned sites provide relatively poor information about motion direction. This situation might occur under at least three possible conditions: (1) sites with strong disparity tuning exhibit weaker or broader direction selectivity than nondisparity-tuned sites, (2) direction preferences are more variable within microstimulation sites that have strong disparity tuning (i.e., direction columns are smaller or less orderly), or (3) neural responses are simply weaker at sites with strong disparity tuning. If disparity-tuned sites indeed provide less-reliable information about the direction of motion, it would be no surprise that the monkey ignored these sites in forming its perceptual decisions. We now describe a battery of analyses to test these possibilities.

Unfortunately, we cannot address the first possibility with our current data set since we did not collect quantitative direction-tuning curves in each experiment due to time limitations (see Materials and Methods). We have, however, examined the relationship between disparity tuning and direction tuning in a large number of separate MU recording experiments conducted in monkey S (*n* = 162) and in three additional monkeys (*n* = 409). Across this unbiased sample of 571 recordings, we find no significant correlation between Disparity Tuning Index (DTI) and Direction Tuning Index (*r* = 0.09, *p* = 0.11; Figure S1). A similar lack of correlation between direction and disparity selectivity was recently reported for a sample of 501 single units recorded in MT (DeAngelis and Uka 2003). We also find no significant correlation (*r* = 0.07, *p* = 0.17) between direction-tuning bandwidth and DTI across our sample of 571 MU recordings, indicating that the sharpness of direction tuning also does not covary with disparity selectivity. These observations, combined with the fact that we only performed microstimulation experiments in the portions of MT with the strongest direction tuning (see Materials and Methods), make us quite confident that the findings shown in Figure 3A do not result from any correlation between direction and disparity tuning in MT.

The last two concerns described above can be addressed directly from the primary data set described in this paper. To evaluate the possibility that direction preferences are more variable within regions of strong disparity tuning (point 2 above), we computed the standard deviation (SD) of directional preferences within a 400-μm region around each microstimulation site. We find no significant correlation between the strength of microstimulation effects and the SD of preferred directions (*r* = −0.04, *p* = 0.68; Figure S2A) and, similarly, no significant correlation between the DTI and the SD of preferred directions (*r* = −0.05, *p* = 0.65; Figure S2B). Thus, the findings shown in Figure 3A do not result from variability in directional preferences. This analysis was performed using estimates of preferred directions from our receptive-field (RF) mapping procedure (see Material and Methods). A separate analysis shows that these estimates have sufficient accuracy and precision for our purposes (Figure S3).

Systematic variations in responsiveness as a function of disparity tuning (point 3 above) can be excluded as a possible explanation for our findings because there is no correlation between the peak response of MU activity and the DTI (*r* = −0.09, *p* = 0.43; data taken from the disparity-tuning curve measured at each stimulation site). Correspondingly, there is no significant correlation between the strength of the microstimulation effects and the peak MU response (*r* = 0.17, *p* = 0.08), and all of the microstimulation effects in Figure 3 were obtained using the disparity that elicited the largest MU response. Similar findings were obtained for each monkey analyzed separately.

Finally, using a dataset of 409 MU recordings and a multiple regression analysis, we also tested for correlations between DTI and several other response properties, including preferred speed, Speed Tuning Index, RF eccentricity, optimal stimulus size, and percentage of surround inhibition. None of these variables was significantly correlated with DTI (*p* > 0.1 for all), indicating that variations in these parameters are also unlikely to account for the results shown in Figure 3A. Collectively, the analyses described above indicate that the failure of microstimulation to elicit behavioral biases at disparity-selective sites cannot be explained by any basic response properties of MT neurons.

### Selectivity of Microstimulation Effects for Binocular Disparity

Although significant microstimulation effects were rare at sites with strong disparity tuning in monkeys S and T, significant effects occurred at a good number of sites with moderate disparity tuning (i.e., DTI > 0.4). At these sites, and at many sites in monkey R, we could ask whether the efficacy of microstimulation varied when the random-dot stimulus was presented at different points along the disparity-tuning curve of the stimulated column.

**Figure 5:**
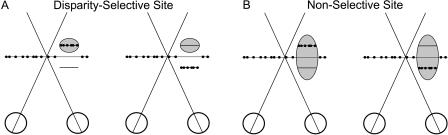
Schematic Illustration of Experiments Designed to Examine Whether Microstimulation Has Disparity-Dependent Effects on Direction Discrimination Each panel is the top-down view of a subject, whose two eyes are represented by the large, open circles. The plane of fixation is indicated by the long horizontal line, along which dots are plotted to represent the stationary, zero-disparity background of random dots. The shaded oval represents the RF—in width and depth—of a hypothetical cluster of MT neurons. (A) Depiction of a disparity-selective site that prefers far disparities (the RF is located behind the plane of fixation). Here, we expect microstimulation to have a significant effect on direction discrimination when dots are presented at the preferred disparity (left) but not when dots are presented at a nonpreferred disparity (right). (B) Depiction of a nondisparity-selective site. The RF is extended in depth, indicating that it has little disparity selectivity. In this case, the effect of microstimulation should not depend on whether dots are presented at either a far (left) or a near (right) disparity.

The logic of this experiment is illustrated for a disparity-selective site in Figure 5A. We hypothesize that neural activity in an MT column that prefers far disparities (shaded oval in 5A) is used primarily to judge direction of motion for planar stimuli at far disparities. Signals from this column should not influence perceptual decisions when the visual stimulus has a near disparity. Accordingly we predict that microstimulation should bias the monkey's choices when dots are presented at the far disparity (Figure 5A, left) and have little or no effect when dots are presented at the near disparity (Figure 5A, right). “Tuned” microstimulation effects of this nature would indicate that motion signals are read out of MT in a disparity-specific fashion. Alternatively, one could imagine that motion signals are pooled across all disparity columns, in which case we should observe nonselective microstimulation effects that are similar for both far and near disparities. For nondisparity-selective stimulation sites (Figure 5B, the receptive field is elongated in depth with respect to the animal's head), we predict that microstimulation will bias the monkey's choices regardless of the binocular disparity given to the visual stimulus.

**Figure 6:**
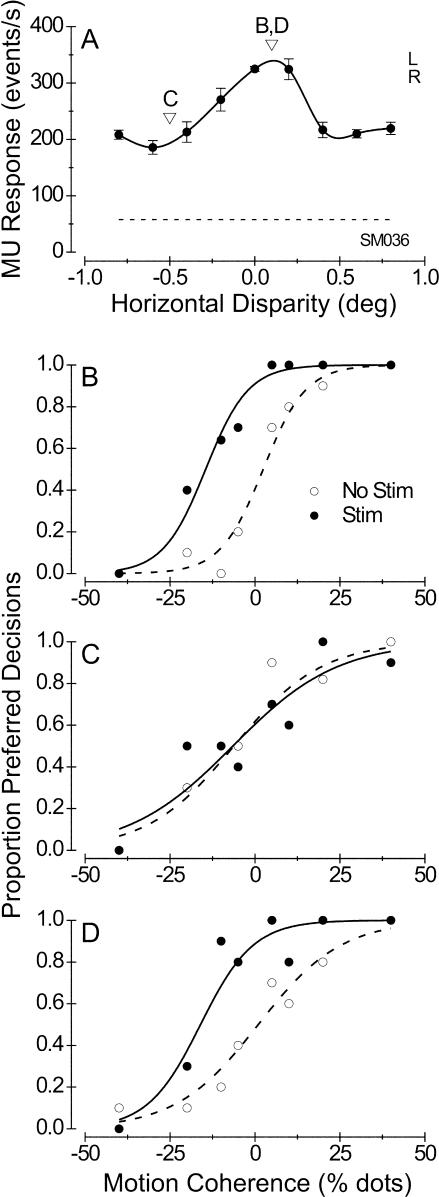
Example of a Disparity-Selective Microstimulation Effect (A) Disparity tuning of MU activity at this stimulation site. Conventions as in Figure 2A. Arrowheads and letters indicate the disparity values used to perform the microstimulation experiments illustrated in (B), (C), and (D). DTI = 0.55. (B) First block of direction discrimination trials, in which dots were presented at the preferred disparity (0.1°). The stimulation psychometric function (filled symbols, solid curve) is shifted well to the left of the nonstimulation function (open symbols, dashed curve) by an amount equivalent to 17% dots (logistic regression, *p* < 0.001), with no corresponding change in the slope of the curve (*p* > 0.9). (C) Second block of discrimination trials, in which dots were presented at a nonpreferred disparity (-0.5°). In this case, the two psychometric functions did not differ significantly in horizontal position (*p* > 0.8) or in slope (*p* > 0.5). (D) Third block of discrimination trials, with dots again presented at the preferred disparity (repeat of [B]). Again, microstimulation produced a leftward shift equivalent to 17% dots (*p* < 0.001). The small increase in the slope of the stimulation psychometric function is not significant (*p* > 0.2).

Figure 6 shows an example of a nicely tuned microstimulation effect. MU activity at this stimulation site exhibited moderate disparity selectivity, with a tuning curve that peaked just to the right of zero disparity (Figure 6A). We performed the microstimulation experiment at two different disparities, denoted by the arrowheads in Figure 6A. In the first block of trials, we presented dots at the preferred disparity (+0.1°), and microstimulation produced a clear leftward shift of the psychometric function that was equivalent to 17% dots (Figure 6B; logistic regression, *p* < 0.001). In the second block of trials, we presented dots at the nonpreferred disparity (−0.5°), and microstimulation exerted no effect whatsoever on the monkey's choices (Figure 6C; logistic regression, *p* > 0.5). To be certain that this effect did not result from some nonstationarity in electrode position, cell responsiveness, etc. (Salzman et al. 1992), we collected a third set of data with dots again presented at the preferred disparity. Again, microstimulation produced a leftward shift of the psychometric function equivalent to 17% dots (Figure 6D; *p* < 0.001). At this stimulation site, therefore, we were able to switch the result from a very substantial effect to no effect and back again simply by manipulating the disparity of the random-dot stimuli.

**Figure 7:**
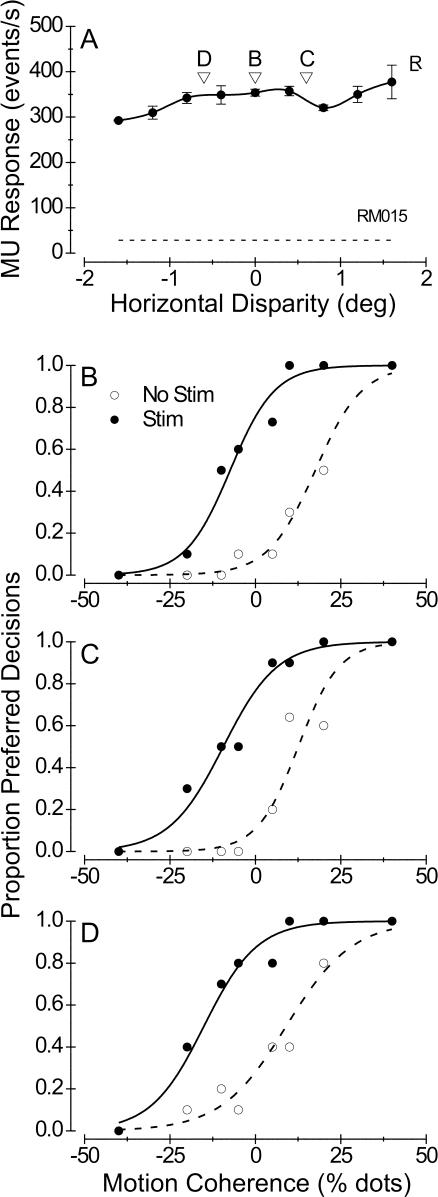
Example of a Nondisparity-Selective Effect of Microstimulation at a Site with Poor Disparity Tuning (A) MU disparity-tuning curve; DTI = 0.27. (B–D) Effects of microstimulation on direction discrimination when dots were presented at disparities of 0°, 0.6°, and −0.6°, respectively. In each case, the leftward shift of the psychometric function is highly significant (logistic regression, *p* < 0.0001) while the slopes were unchanged (*p* > 0.4).

Figure 7 depicts data from experiments performed at a nondisparity-selective site. The MU activity at this site exhibited little selectivity for binocular disparity, although the tuning was marginally significant (Figure 7A; ANOVA, *p* = 0.025). We chose three different disparities at which to perform the direction discrimination task: 0°, 0.6°, and −0.6°. Figure 7B–7D show the effects of microstimulation on direction judgments at these three different disparities. In each case, microstimulation induced a significant leftward shift of the psychometric function (logistic regression, *p* < 0.0001), with no corresponding change in slope (*p* > 0.4).

The individual example sites in Figures 6 and 7 conform well to the predictions of our hypothesis outlined in Figure 5. We observed considerable variation across the population of experiments, however, so we quantified the disparity selectivity of each microstimulation effect in order to evaluate statistical trends in the population. We performed this analysis on 65 out of 102 data sets for which we had applied microstimulation at both the preferred and nonpreferred disparities, and for which the effect of microstimulation was significant (*p* < 0.05) for at least one of the two disparities. We computed a Microstimulation Selectivity Ratio (MSR) as follows:



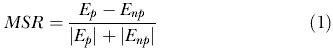



where *E_P_* is the effect of microstimulation when dots are presented at the preferred disparity, and *E_NP_* is the effect when dots are presented at the nonpreferred disparity. This index is a standard contrast measure, except that the quantities in the denominator are absolute values. This formulation was necessary to keep the index bounded between −1.0 and 1.0.

**Figure 8:**
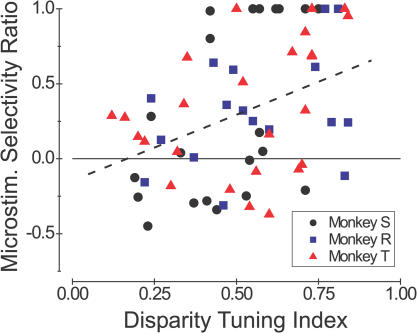
Quantitative Summary of the Disparity Selectivity of Microstimulation Effects The ordinate is the MSR, which was computed from the leftward shifts of the psychometric function measured at both the preferred and nonpreferred disparities (Equation 1). The abscissa is the DTI of MU activity at each stimulation site. Data are shown for 65/102 stimulation sites for which a significant effect of microstimulation was observed at either the preferred or nonpreferred disparity. Results from monkeys S, R, and T are shown as black circles, blue squares, and red triangles, respectively. Data points with an MSR equal to1.0 correspond to cases where there was a leftward shift of the psychometric function at the preferred disparity and a rightward (i.e., null-direction) shift, or no shift, at the nonpreferred disparity. The dashed line shows the best linear fit to the data (ANCOVA, *r* = 0.37, *p* < 0.005).

Figure 8 shows the MSR plotted against the DTI, with different symbols denoting data from the three monkeys. To analyze the relationship between MSR and DTI without confounding possible effects of monkey differences, we performed an analysis of covariance (ANCOVA) with DTI and monkey identity as factors. This analysis reveals a significant correlation between MSR and DTI (ANCOVA, *r* = 0.37, F(1,61) = 9.9, *p* < 0.005), with no significant differences between the three monkeys (F(2,61) = 0.14, *p* > 0.8).

Thus, as hypothesized (see Figure 5), microstimulation generally exerted selective effects at sites with strong disparity tuning, and nonselective effects at sites with poor tuning. Although this relationship between MSR and DTI was not very strong (as evidenced by the large scatter of points in Figure 8), almost all of the strongly selective microstimulation effects (MSR > 0.5) occurred at sites with moderate to strong disparity tuning (DTI > 0.4). The upper left corner of Figure 8 is notably unpopulated, indicating that selective effects of microstimulation did not occur at poorly disparity-tuned sites. Possible reasons for the variability in Figure 8 will be discussed below.

## Discussion

Using microstimulation to probe the link between neuronal activity and behavior, we have tested whether the contribution of MT neurons to direction discrimination depends on their disparity selectivity. This work addresses the general question of how neurons that are tuned to multiple stimulus dimensions contribute to behavior in situations where one or more of these stimulus dimensions are task-irrelevant. Relatively little is currently known about how the responses of sensory neurons are pooled by decision mechanisms (see Shadlen et al. 1996) and how the demands of a particular task alter the pooling strategies that are used. The present study provides new insights into these issues. Our first main finding is that the strength of tuning for binocular disparity (an irrelevant variable in the direction discrimination task) accounts for a substantial proportion of variance in the strength of microstimulation effects (48% of variance for monkey S, 27% for monkey T). Two of our three monkeys relied mainly on nondisparity-selective sites for performing the direction discrimination task, even though the stimulus was tailored to the disparity preference of all sites. Our second main finding is that the efficacy of microstimulation is reduced when the stimulus disparity is adjusted to be suboptimal for neurons at the stimulation site. Thus, to the limited extent that our monkeys made use of signals from disparity-selective neurons, they did tend to monitor more closely neurons with tuning properties that were matched to the stimulus. This latter finding can be viewed as a generalization to three dimensions of the previous result that microstimulation effects were reduced by moving the visual stimulus out of the RF of the stimulated neurons (Salzman et al. 1992).

### Effects of Disparity Tuning Strength: Local Circuit Properties, Connectivity, or Task Strategy?

How can we explain the finding (see Figure 3A) that regions of MT that are selective for both direction and disparity generally do not contribute to direction discrimination, despite the fact that stimulus parameters were always optimized for the disparity tuning of these neurons? One relatively uninteresting possibility is that unknown cellular or circuit properties specific to disparity-sensitive columns limit the efficacy of microstimulation. For example, disparity-selective regions of MT, which tend to be segregated from nonselective regions (DeAngelis and Newsome 1999), might have different biophysical properties, metabolic properties, local connectivity, or patterns of afferent input. Such factors are unlikely to account for our results, however, given the data illustrated for monkey T in Figure 4. Because columns with large disparity-tuning indices generally fail to yield effects in the direction discrimination task but yield good effects in the disparity discrimination task, we can reject explanations based on factors endogenous to local regions of MT.

A second possibility is that the output connections of disparity-selective and nonselective regions of MT have different targets, such that decision mechanisms for motion receive input from nondisparity-selective portions of MT whereas decision mechanisms for depth receive input from disparity-tuned regions. Experiments have not been done to test this hypothesis, so we cannot rule it out. One argument against this idea, however, is that one of the three monkeys (monkey R) did not show a dependence of microstimulation effects on disparity selectivity (see Figure 3B). Thus, for anatomical projections of MT to explain our findings, we would have to assume that both disparity-selective and nonselective regions of MT project to decision mechanisms for motion perception in monkey R, but not in the other two animals. Experiments involving tracer injections into regions of MT chosen for strong versus weak disparity tuning would be valuable for examining this possibility.

A third possibility, which we favor, is that our findings reflect the strategy that each monkey adopted for reading out motion signals from MT during the extended period of training on the task. In this scenario, all regions of MT could project to decision mechanisms for both motion and depth, but the relative weights of the connections would vary with the animal's task strategy. This would allow the read-out strategy to be altered rapidly based on the demands of the task. In our experiments, one strategy for performing the task would be to extract motion signals from all MT columns with the appropriate direction selectivity and spatial RF, regardless of their disparity selectivity. This strategy would entail pooling signals from many columns, including those with unfavorable signal-to-noise ratios due to their poor responsiveness to stimuli of nonoptimal disparity.

A second strategy, which could yield better performance, would be to monitor primarily columns that are maximally activated by the stimulus, but this would entail pooling responses from columns with different disparity preferences when the stimulus disparity changed. Thus, some sort of complex “switching” would be required to route information to the decision process from the set of columns optimal for each experiment. A third, and perhaps the simplest, strategy would be to monitor motion signals only from the nondisparity-selective portions of MT; these columns would respond well to all stimulus disparities, providing a good signal-to-noise ratio for all stimulus sets on which the monkey was trained. This strategy offers the further advantage that one can monitor the same set of columns for all stimulus conditions in our task. Given that correlated noise among neurons limits the benefits of pooling across large populations of neurons (Britten et al. 1992; Shadlen et al. 1996), this last strategy might yield performance almost as good as that obtained by monitoring all columns that are strongly activated by a particular disparity.

If monkeys were to adopt the simple strategy of monitoring only the nondisparity-selective regions of MT, then the microstimulation results shown in Figure 3A (monkeys S and T) would be expected. The very different results seen for monkey R (see Figure 3B) would not be the result of distinct output projections from disparity-selective and nonselective regions of MT, but rather would indicate that synaptic weights were dynamically modulated in monkey R to route information to decision circuits from all columns that were well activated by the stimuli. This conclusion is supported by the data shown in Figures 3B and 8, which together show that monkey R monitors direction signals from disparity-selective columns provided that the stimulus disparity matches the disparity preference of the neurons. Indeed, our finding that monkey R had a significantly lower psychophysical threshold than the other two animals is fully consistent with the task strategy suggested by our microstimulation results. In future experiments, it will be interesting to find ways to alter the monkeys' task strategies while using microstimulation to probe the contributions made by a single column of MT neurons.

### Disparity Tuning of Microstimulation Effects: Origins of Variability

We found a statistically significant, but relatively weak, dependence of microstimulation effects on the difference between the preferred disparity of MT neurons and the stimulus disparity (see Figure 8). What accounts for the relatively large variability in these data? For monkeys S and T, microstimulation effects were usually weak at disparity-selective sites, and this could contribute to the scatter seen in Figure 8. If this were the case, then the correlation in Figure 8 should be stronger for monkey R, given that microstimulation of disparity-selective sites was usually quite effective in this animal. Inspection of Figure 8 reveals that this is not the case, however. In fact, the correlation coefficient between MSR and DTI (see Figure 8) was stronger for monkey S (*r* = 0.55, *p* < 0.01) than for monkey R (*r* = 0.36, *p* = 0.15).

Another possible source of variability in Figure 8 involves the fact that we tested the effects of microstimulation in different blocks of trials for different disparities (see Materials and Methods). Given that microstimulation effects frequently wane as a function of time (Salzman et al. 1992) and are sensitive to small perturbations in electrode position (Murasugi et al. 1993), this block design would be expected to add noise to the population data. Another likely source of variability involves the selection criteria for microstimulation sites. We attempted to center our electrode in the midst of a region of constant direction tuning, but we did not select sites based on the consistency of disparity tuning within the neighborhood of the electrode. Thus, even when MU activity at the stimulation site was strongly disparity tuned, our electrode may have been positioned close to a boundary between a near column and a far column, or simply within a region where disparity tuning was changing rapidly (DeAngelis and Newsome 1999). This may have allowed microstimulation to activate a population of neurons that responded well to both stimulus disparities in some cases.

Considering these likely sources of variability, the fact that we see a significant overall effect in Figure 8 provides solid evidence that monkeys do monitor more closely columns of neurons with stimulus preferences that match the prevailing stimulus parameters. It is worth noting that our ability to observe this effect may have been aided by the blocked design that we employed. Because the stimulus disparity was fixed within a block of trials, monkeys could selectively monitor MT columns tuned to that disparity. In contrast, microstimulation effects might be less disparity selective if the stimulus disparity varied from trial to trial, such that the animal was uncertain about which disparity columns to monitor.

### General Implications

Many of the standard experimental approaches in systems neuroscience (e.g., single-unit recording, optical imaging, functional MRI) find their utility in exposing correlations between neuronal activity and external stimuli or behavioral states. Of course, finding signals that are correlated with behavior does not prove that those signals underlie the behavior. The value of electrical microstimulation, reversible inactivation, and lesion techniques is that they can establish causal links between neural activity and behavior. In this study, we only microstimulated at sites in MT that had strong directional selectivity; thus, one might assume that all sites would be equally likely to contribute to performance of the direction discrimination task. The central finding of this study is that the contribution of MT direction columns to task performance is modulated by the tuning of the neurons to a stimulus variable that is irrelevant to completion of the task. Thus, even within a single area of the brain, the causal linkage between neurons and behavior may depend on uncontrolled stimulus dimensions, and may be determined by unexpected factors such as task strategy. This result highlights the importance of causal techniques for studying the neural basis of behavior, and suggests that microstimulation studies may be able to reveal how high-level task strategies modulate the read-out of neuronal signals from topographic maps in the brain.

## Materials and Methods

### 

Our standard procedures for surgical preparation, training, and electrophysiological recording from rhesus monkeys *(Macaca mulatta)* are described elsewhere (Britten et al. 1992). In addition, extensive details of our microstimulation techniques have been published elsewhere (Salzman et al. 1992). Here, we briefly describe our methods, focusing on aspects that are particularly relevant to the present study.

#### Surgical preparation

Three adult macaques were used in this study (two males and one female), all of which had previously been subjects in other studies in the laboratory. Each animal had a scleral search coil implanted in at least one eye (monkey S had coils in both eyes) to allow monitoring of eye position. In addition, each subject was equipped with a head restraint post and a stainless-steel recording chamber that was positioned over the occipital cortex. Electrodes were introduced into the visual cortex through a transdural guide tube that was positioned within a square array of grid holes at 1-mm intervals (Crist et al. 1988).

#### Visual stimuli and tasks

All visual stimuli used in this study were dynamic random-dot patterns presented on a standard 21-in. color display (Sony 500PS, Sony Corporation, New York, New York, United States). The display subtended 39° × 29° at the viewing distance of 57 cm and was refreshed at a rate of 100Hz. The visual stimuli were generated by a Cambridge Research Systems VSG2/3 board (Cambridge Research Systems Ltd., Rochester, United Kingdom) that was housed in a dedicated PC. Stereoscopic presentation was achieved through the use of ferroelectric shutters (Displaytech, Inc., Longmont, Colorado, United States) that were switched in antiphase for the two eyes. Left and right half-images were presented on alternate video frames, and the shutters were synchronized to the vertical refresh, thus exposing each eye to the appropriate visual stimulus on alternate frames. With this technique, the quality of stereo separation is limited mainly by phosphor persistence. Thus, random-dot stimuli were always presented using the red gun only, since the red phosphor has a much faster decay than either the green or blue phosphors. We achieved a contrast ratio of approximately 40:1 (“open” eye:“closed” eye) using this approach, and “ghosting” artifacts were barely visible, even under dark-adapted conditions.

Monkeys performed two separate tasks in these experiments: a visual fixation task, and a direction discrimination task. In the visual fixation task, a small, yellow fixation point (FP) appeared to begin each trial, and the monkeys were required to maintain fixation within a 2° × 2° or 3° × 3° electronic window, centered on the fixation target, until the fixation target was extinguished. The monkeys received a liquid reward for successful fixation, typically 0.1–0.15 ml of water or juice. If the monkey broke fixation before the end of a trial, the trial was aborted, the data were discarded, and the monkey was not rewarded. During the fixation period, a bipartite random-dot stimulus was presented for 1.5 s. It consisted of a central, circular patch of coherently moving dots that could be presented with variable binocular disparity, and which covered the receptive field of the MT neurons under study. To assist the monkey in maintaining binocular convergence on the FP, we filled the remainder of the visual display with zero-disparity dots that were randomly repositioned every fourth video frame (25 Hz), thus producing a twinkling, zero-disparity background. Each dot was approximately 0.1° in size. Dot density was 32 dots/(deg^2^-s) for the central patch and 8 dots/(deg^2^-s) for the background.

In the direction discrimination task (see Figure 1), each trial also began with the presentation of a FP. Once the monkey fixated, a bipartite random-dot pattern again appeared. The central, circular patch had variable motion coherence. On each video frame, a fraction of the dots (“signal” dots; filled in Figure 1A) moved coherently in either the preferred or null direction of the MT neurons under study. The remaining dots in this center patch (“noise” dots; unfilled in Figure 1A) were replotted at random positions in each video frame. Thus, the strength of the motion signal (percent coherence) is determined by the percentage of signal dots in the display (see Britten et al. 1992 for additional details). Signal dots moved in the preferred direction on one-half of all trials and in the null direction on the remaining trials (randomly interleaved). Outside of the center patch, the remainder of the video display was filled with stationary zero-disparity dots to serve as a background. The random-dot motion stimulus ran for 1 s, after which both the FP and the dots disappeared. Two disk-shaped targets then appeared, aligned with the axis of stimulus motion, and the monkey indicated its perceived direction of motion by making a saccade to the target toward which the signal dots moved. Again, the monkeys received liquid rewards for correct choices. Incorrect choices resulted in no reward and a brief time-out period between trials. Dot size and density were as described above for the fixation task.

#### Microstimulation

On one-half of the direction discrimination trials, selected at random, electrical microstimulation was applied during presentation of the random-dot stimulus. The microstimulation current was delivered through a stimulus isolation unit (Bak Electronics, Inc., Mount Airy, Maryland, United States) operating in constant-current mode. The current was a train of biphasic pulses with a frequency of 200 Hz and an amplitude of 20 μA. Each biphasic event consisted of a 200-μs cathodal pulse followed by a 200-μs anodal pulse, with a 100-μs gap between the two. Microstimulation parameters were chosen to elicit robust perceptual biases but were well below the current and frequency levels at which stimulation has been shown to flatten the slope of the psychometric function (Murasugi et al. 1993). Microstimulation was applied through the same parylene-coated tungsten electrode (MicroProbe, Inc., Carlsbad, California, United States) that was used to record unit activity in MT.

#### Selection of microstimulation sites

We searched for candidate microstimulation sites by examining the tuning properties of MU activity at regular intervals of 100 μm along electrode penetrations through MT. At each recording site, we rated the strength of direction selectivity on a scale from 1 to 3 (3 = strongest tuning), and we carefully estimated the preferred direction of motion (see Figure S3 regarding the accuracy and precision of these estimates). We accepted a site for microstimulation when there was a span of at least 300 μm in which direction selectivity was consistently rated a 3 and the preferred direction of motion varied by no more than 45°. Disparity selectivity had no bearing on our selection of stimulation sites in this study; thus, our sample of stimulation sites should be unbiased in terms of disparity tuning. Once a suitable span of direction tuning was identified, we retracted our electrode to approximately the middle of the span and began quantitative testing.

#### Experimental protocol

At each identified microstimulation site, we performed the following battery of tests. (1) First, we carefully mapped the MU RF of the MT neurons by dragging a small patch of moving dots (100% coherence) through the RF with a pointing device. Spike densities were plotted on a Cartesian map of visual space during this process to facilitate visual mapping of the RF. In addition, we mapped the direction and speed selectivity of the neurons by moving a cursor throughout a polar direction-speed domain while spike densities were again plotted on the screen. From this procedure, we determined the location and size of the MU RF, as well as the preferred direction and speed of motion. We also estimated the range of disparities over which the neurons were selective, and these parameters were then used in subsequent quantitative tests. (2) We next measured a disparity-tuning curve for MU activity at the identified stimulation site, while the monkey performed a block of fixation trials. Nine evenly spaced disparities were typically tested within the disparity range determined from our initial qualitative probing (e.g., see Figure 2A and 2C). Monocular control conditions were also included, and all trial conditions were block randomized and repeated four to five times. For MU responses in MT, this number of repetitions proved more than adequate to obtain tuning curves with small error bars. The central patch of dots (which varied in disparity) was adjusted to be slightly larger than the MU RF, and all dots within this central patch moved coherently in the neurons' preferred direction of motion (at the preferred speed). Note that in a previous study (DeAngelis and Newsome 1999), we established that these MU measurements of disparity tuning in MT reliably predict the disparity tuning of single units within the neighborhood of the electrode tip. Due to limitations of recording time, we did not measure a quantitative direction-tuning curve at each microstimulation site. (3) We next applied microstimulation during blocks of trials in which the monkey performed the direction discrimination task (see Figure 1) along the preferred-null axis of motion. Motion coherence was varied from trial to trial within a range of values that bracketed the psychophysical threshold of each animal, as determined during training. At each site, we collected at least two blocks of discrimination trials: one at the preferred disparity and one at the nonpreferred disparity. The order of these two blocks was counterbalanced across experiments, and statistical analyses revealed no significant effects of block order on any of our results (ANCOVA, *p* > 0.3). Whenever possible (e.g., see Figure 6D), we performed a third block of trials at the same disparity tested in the first block. For sites with no clear disparity preference at all (as measured on-line), the choice of disparities for the direction discrimination task was arbitrary. In these cases, we typically performed three blocks of trials with disparities of (approximately) −0.5°, 0°, and 0.5°, although the order in which these disparities were presented was varied from site to site.

During training, we attempted to interleave two different disparities within a single block of direction discrimination trials. Although this approach would clearly be superior to a blocked design in some respects, we found that interleaving the disparities resulted in poorer discrimination performance because the monkeys' choices were biased by stimulus disparity when the motion signal was weak. We therefore settled for the block design described above.

#### Data collection

Extracellular recordings were made with tungsten microelectrodes (impedance typically 0.5–1.0 MΩ; MicroProbe, Inc.). Neural signals were amplified, filtered (0.5–5.0 kHz), and discriminated using conventional electronics (Bak Electronics, Inc.), and event times were stored on magnetic disk with 1 ms resolution. To record MU activity, we simply set the threshold level of our window discriminator to approximately 1–2 SD above the noise level. Thus, a MU event was defined as any deflection of the analog signal that exceeded this threshold. Since the absolute frequency of the MU response depends heavily upon the event threshold, we attempted to achieve a consistent response magnitude from site to site by adjusting our event threshold such that the spontaneous activity level was in the range from 50 to 100 events/s. This setting typically yielded peak MU responses in the range of 300–500 events/s (mean 378.5 ± 78.3 SD).

Horizontal and vertical eye-position signals were low-pass filtered with a cutoff frequency of 250Hz, sampled at 1 kHz, and stored to disk at 250 Hz.

#### Data analysis

To construct disparity-tuning curves, we computed the firing rate for each trial during the 1-s stimulus presentation, and we plotted the mean firing rate (± SE) as a function of the horizontal disparity. Smooth curve fits to disparity-tuning curves were achieved using a cubic spline interpolation. To quantify the strength of disparity tuning at each stimulation site, we computed the DTI as follows:



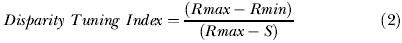



where *Rmax* denotes the response to the preferred disparity, *Rmin* denotes the response to the antipreferred disparity, and *S* indicates the spontaneous activity level. Values larger than unity can occur if *Rmin* is less than *S*. For the quantification of direction-tuning strength (see Figure S1), a Direction Tuning Index was defined in an identical fashion.

We analyzed behavioral data by computing the proportion of preferred decisions that the monkey made for each different combination of motion coherence and direction, where a preferred decision is defined as that in favor of the preferred direction of MU activity at a particular microstimulation site. This proportion was plotted as a function of the signed motion-coherence variable (see Figure 2B), where positive coherences correspond to motion in the preferred direction and negative coherences to motion in the antipreferred direction. The statistical significance of microstimulation effects was determined using a logistic regression analysis, as described by Salzman et al. (1992).

## Supporting Information

Figure S1 Relationship between Strength of Direction Tuning and Strength of Disparity Tuning in MT Data are shown from 571 MU recordings (162 from monkey S, shown in red, and 409 from three additional animals, shown in black) in which we obtained quantitative measurements of both direction tuning and disparity tuning. There is no significant correlation between Direction Tuning Index and Disparity Tuning Index (DTI) across the sample. Note also that the data from monkey S overlap completely with the data from the other animals, indicating that monkey S was not unusual.
(358 KB EPS).

Figure S2 Analysis of Direction Preference Variability at Microstimulation Sites in Monkey S and Monkey T Monkey S is shown in black; monkey T in red.
(A) The strength of the microstimulation effect is plotted against the SD of direction preferences within a 400-μm window centered on each stimulation site (five recording sites, 100 μm apart). There is no significant correlation between these variables, indicating that variability in direction preferences (within the observed range) did not determine the efficacy of microstimulation. Note, however, that all stimulation sites were chosen to have a small range of preferred directions; we did not apply microstimulation at locations in MT where the direction preference changed rapidly over short distances.
(B) There is also no significant correlation between the DTI of MU activity at each stimulation site and the SD of direction preferences. This shows that disparity-selective microstimulation sites did not have larger variations in direction preferences.
(216 KB PS).

Figure S3 Comparison of Direction Preference Estimates Obtained from Post Hoc Gaussian Fits of Direction-Tuning Curves Versus Online Estimates of MT Preferred Directions See Materials and Methods. Data were obtained from 409 single units in MT of three animals that were not part of the present study. For 68% of neurons, the two direction preference estimates differ by less than 20°. By comparison, the mean directional bandwidth (full width at half-maximal height) for this population of neurons was 121° ± 54° SD; hence, the error in hand-mapped estimates of direction preference is quite small relative to the breadth of tuning.
(320 KB EPS).

## Abbreviations

DTI: Disparity Tuning Index
FP: fixation point
MSR: Microstimulation Selectivity Ratio
MT: middle temporal
MU: multiunit
RF: receptive field
SE: standard error
SD: standard deviation
